# Combined Liquid-Based Cytology and Conventional Smear Provides Better Sensitivity and Adequacy Rates After Endoscopic Ultrasound-Guided Tissue Acquisition of Abdominal Masses: A Systematic Review and Meta-Analysis

**DOI:** 10.3390/jcm14186685

**Published:** 2025-09-22

**Authors:** Marie Anne Engh, Brigitta Teutsch, Alexander Schulze Wenning, Tamás Kói, Péter Hegyi, Bálint Erőss

**Affiliations:** 1 Centre for Translational Medicine, Semmelweis University, 1085 Budapest, Hungary; 2 Institute for Translational Medicine, Medical School, University of Pécs, 7624 Pécs, Hungary; 3 Department of Radiology, Medical Imaging Centre, Semmelweis University, 1082 Budapest, Hungary; 4 Department of Stochastics, Institute of Mathematics, Budapest University of Technology and Economics, 1111 Budapest, Hungary; 5 Institute of Pancreatic Diseases, Semmelweis University, 1083 Budapest, Hungary; 6 Translational Pancreatology Research Group, Interdisciplinary Centre of Excellence for Research Development and Innovation, University of Szeged, 6720 Szeged, Hungary

**Keywords:** cytology, EUS, pancreatic masses, abdominal masses

## Abstract

**Background and Aims:** Endoscopic ultrasound (EUS)-guided fine-needle aspiration (FNA) or biopsy (FNB) is the standard method for diagnosing abdominal masses, but sample inadequacy and diagnostic accuracy remain challenges. Conventional smear (CS) and liquid-based cytology (LBC) are standard processing methods, yet their comparative effectiveness and potential combined benefit remain unclear. We performed a systematic review and meta-analysis to evaluate and compare the diagnostic performance and adequacy of CS, LBC, and their combination. **Methods:** A systematic search was conducted in Medline, Embase, and CENTRAL on 17 November 2024. Studies comparing CS, LBC, or their combination following EUS-FNA/FNB for abdominal masses were included. Diagnostic parameters, including sensitivity, specificity, accuracy, and inadequacy rates, were extracted and analyzed. Methodological quality was assessed using QUADAS-2. **Results**: 16 studies (2128 patients) were included. Sensitivity for pancreatic masses was 71.4% (CI: 62.9–78.7) for CS, 74.7% (CI: 64.3–82.8) for LBC, and 86.2% (CI: 82.4–89.3) for combined methods (*p* = 0.001). For all abdominal masses, sensitivity was 76.3% (CI: 67.9–83.0) for CS, 73.6% (CI: 65.6–80.2) for LBC, and 88.0% (CI: 84.0–91.2) for combined methods (*p* ≤ 0.006). Specificity was nearly 100%. Inadequacy rates were lowest for combined methods (1.5%, CI: 0–36.2), when compared to LBC (7.7%, CI: 2.7–20.4) and CS (4.4%, CI: 2.4–7.9). Moderate bias risk was noted, primarily due to incorporation bias. Domain 3 (reference standard) of QUADAS was uniformly moderate-risk across studies. **Conclusions**: Combining CS and LBC methods improves diagnostic sensitivity and reduces sample inadequacy after EUS-guided tissue acquisition for abdominal masses, particularly pancreatic lesions. Clinical guidelines should consider recommending the combined approach to enhance diagnostic yield and clinical outcomes.

## 1. Introduction

Cancerous lesions in and around the gastrointestinal (GI) tract represent a major global health burden, accounting for 26% of global cancer incidence burden and 35% of all cancer-related deaths [1]. Notably, pancreatic cancer has some of the lowest survival rates, with pancreatic cancer five-year survival ranging from 10% to 18% depending on the country [2]. In the case of pancreatic cancer, this is largely due to late-stage diagnosis, as lesions are often detected after the disease has progressed, leading to a poor prognosis.

With advancements in minimally invasive techniques, endoscopic ultrasound (EUS)-guided fine-needle aspiration (FNA) or biopsy (FNB) has become the preferred method for obtaining pathological samples from peri-GI lesions [3]. Despite being less invasive, these procedures still carry risks [4]. Moreover, sample adequacy is not guaranteed, with reported success rates ranging from 60% to 90%, depending on factors such as rapid on-site examination, needle type used, and other factors. This problem has prompted ongoing efforts to improve sample adequacy and diagnostic accuracy through innovations in equipment, technique, and processing methods [5,6].

One such area of focus is the optimal processing of cytology specimens. The Conventional Smear (CS) technique and Liquid-Based Cytology (LBC)—the latter of which is the gold standard in fields such as gynecology—are two primary approaches. Several studies, including meta-analyses, have compared these methods, though results have varied due to methodological differences and inconsistent findings [7,8,9]. Conflicting results across earlier reviews reflect methodological heterogeneity: outcome measures (diagnostic parameters vs. inadequacy), comparators (single method vs. CS + LBC), and study designs. In addition, ROSE availability, the LBC technique (filtration vs. precipitation), and paired vs. unpaired comparisons varied between evidence bases.

Current international guidelines reflect this uncertainty. The European Society for Gastrointestinal Endoscopy (ESGE) [10] recommends a combination of CS and LBC for pancreatic EUS samples, though this is based on low-quality evidence. In contrast, the Korean Society for Gastrointestinal Endoscopy (KSGE) [11] acknowledges the importance of cytology method selection but does not issue a specific recommendation.

In this study, we aim to systematically review and meta-analyze existing data by comparing CS, LBC, and their combination in terms of diagnostic yield and sample adequacy to provide a more substantial evidence base for future guidelines.

## 2. Methods

This systematic review and meta-analysis has been conducted according to the guidelines of the Cochrane Collaboration [12] and is reported following the PRISMA 2020 guideline [13]. The PRISMA checklist can be found in the Supplementary Material. The protocol of this study was fully adhered to and was registered on PROSPERO (registration number: CRD42024612112). An additional analysis was performed to assess the inadequacy rate of the different methods.

### 2.1. Eligibility Criteria

Studies including patients undergoing EUS-guided FNA or FNB for an abdominal lesion (pancreatic, gastrointestinal, or other) were eligible for inclusion. Only studies that reported diagnostic parameters of both liquid-based cytology and conventional smear were included to minimize confounders in the comparator. Due to a limited number of studies predicted, studies reporting on the combined diagnostic value of liquid-based cytology and conventional smear were eligible for inclusion regardless of whether the two cytology methods were also separately reported. Attempts were made to deduce the combined diagnostic value from papers that did not report it through their degree of agreement, assuming that one positive test would mean a positive by combination.

Both indexed journal articles of any study design and conference abstracts were eligible for inclusion, provided they contained the information necessary for analysis.

### 2.2. Information Sources and Search Strategy

The systematic search was conducted on 17 November 2024, in Medline (via PubMed), Embase, and CENTRAL. The search strategy included a domain for the abdomen/GI/pancreas, a domain for tissue acquisition, and a domain for the type of tissue preparation. The exact search key can be found in the Supplementary Material (Supplementary Text S1).

References of the included studies were screened for further eligible studies, and papers citing the included studies were searched on 17 November 2024, using the citationchaser [14] tool.

### 2.3. Selection Process

The selection was performed by two independent review authors (MAE and ASW) after the duplicates were removed. Records were first screened by title and abstract, then by full text. Disagreements were resolved by discussion. Cohen’s kappa was used to quantify the degree of interrater agreement. Citing papers and references were handled as two separate pools of records, and selection was performed in the same two stages.

### 2.4. Data Collection Process

Two independent reviewers (MAE, ASW) extracted data in a prospectively designed Excel sheet. The primary investigator (MAE) compared the data and any points of contention resolved by a consensus. Data were sought on the study design, years of patient enrollment, study population (type of lesions, age, and gender), sampling procedure (needle type), reference, number of benign and malignant cases, and outcomes. The outcome variables sought were true positives, false negatives, true negatives, and false positives. Where available, we also extracted accuracy, sensitivity, specificity, and positive and negative predictive values with confidence intervals (CIs).

The case numbers were reverse engineered from the available information if only sensitivity, specificity, and accuracy were given without CIs, but the total number of benign and malignant cases were known.

If diagnoses were given as a table with the different pathological definitions according to the Papanicolau Society of Cytopathology [15], malignant and suspicious were considered malignant, and all others were considered benign in the extraction.

In the primary analysis, to increase homogeneity, studies were only included if the definition of malignancy was either “malignant and suspicious”, or case numbers were extractable in accordance with the definition.

Two analyses were done: First, studies on solid pancreatic masses were included, and second, all types of abdominal masses were included.

### 2.5. Study Risk of Bias Assessment

The risk of bias was assessed using the QUADAS-2 tool [16] by two independent reviewers (MAE, ASW) and discussed internally to resolve conflicts. Results were visualized using the robvis tool [17].

### 2.6. Synthesis Methods

Statistical analyses were performed using R statistical software (version 4.1.2) and the R script of the online tool described by Freeman [18]. Random-effect meta-analysis was applied for each outcome. A *p*-value of less than 0.05 was considered significant for all statistical analyses.

Two by two contingency tables were directly extracted or calculated from the studies containing true positive, false positive, false negative, and true negative values. Several diagnostic measures can be calculated from these numbers. As usual in diagnostic meta-analysis, we only meta-analyzed the sensitivity and specificity. They are the preferred outcomes since they do not depend on the proportion of the malignant cases. To provide better insight, using the pooled sensitivity and specificity, we calculated accuracy, PPV and NPV, assuming several different malignancy prevalence assumptions. In the case of sensitivity and specificity, only the corresponding random effects can be correlated; the within-study correlation between them is zero. In contrast, when a study reports results corresponding to more than one diagnostic tool evaluated on the same patients, there is also within-study correlation in the data. The Clubsandwich R package (version 4.1.2) provides a robust tool for handling within-study correlation but only works with linear meta-analysis models. For this reason, instead of the usual mixed-effect logistic regression-based bivariate approach of Reitsma and Chu [19,20], we used a linear model on the logit transforms of sensitivity and specificity, and we had to analyse them separately. The fact that almost all specificities were precisely one further justifies the separate analyses.

The performances of the conventional smear, LBC, and combined methods were frequently evaluated in the same population within the studies. For this reason, for the logit transformed sensitivity, we constructed a three-dimensional model using the rma.mv() function of the metafor R package. To circumvent the problem caused by the unknown correlations, we supplemented the method with the robust approach of Pustejovsky [21], implemented in the coef_test() function of the clubSandwhich R package. Moreover, we repeated the approach under several within-study correlation assumptions. All sensitivity runs provided similar *p*-values. For the specificity, this approach was not feasible. Hence, we calculated pooled specificity using the generalized mixed-effect approach of Stijnen et al. [22]. We used a mixed approach to meta-analyze the inadequate sample ratio. Namely, due to the presence of studies with no inadequate sample, to calculate pooled results within the subgroup, we used the methodology of Stijnen et al., while after adding 0.1 to the zero frequencies (continuity correction), we followed the robust approach described above to generate *p*-values.

We visualized the results on forest plots. Heterogeneity was assessed by calculating the I^2^ measure and its confidence interval. In the case of the robust multivariate sensitivity analysis, the I^2^ statistics were calculated for each method separately using univariate methodology.

For the reasons mentioned above, we performed publication bias analyses only for sensitivity. In the case of prevalence, the effect size and the standard error are dependent. For this reason, following the suggestion of Hunter et al. [23], we created a modified funnel plot to access the publications bias visually: on the y-axis, we plotted the study size instead of the standard error. Moreover, instead of Egger’s test, we used Peters’ test [24] to test whether publication bias is present. We assessed publication bias separately in each subgroup with at least 10 studies.

## 3. Results

### 3.1. Search and Selection

**Figure 1:**
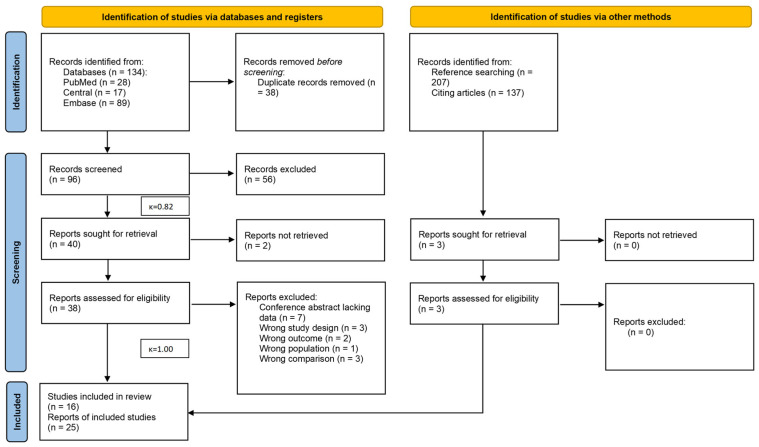
PRISMA 2020 flowchart representing the study selection process.

Our search strategy identified 134 records, of which 22 reports [25,26,27,28,29,30,31,32,33,34,35,36,37,38,39,40,41,42,43,44,45,46] (13 studies) were eligible for inclusion. Three further studies [47,48,49] were identified from the reference and citation search. All the studies included focused exclusively on FNA. The entire selection process is detailed in the PRISMA flowchart in Figure 1. One paper [50] was first deemed eligible, then excluded, as the type of cytology was part of a compound change in sample processing, which would likely represent significant confounding while following a different definition of malignancy than that employed in this review.

### 3.2. Basic Characteristics of Included Studies

The baseline characteristics, including patient characteristics, methods used for cytology, needle designs used, and the type of mass, are detailed in Table 1.

### 3.3. Sensitivity and Specificity

Eleven studies [25,27,32,33,38,41,42,45,46,47,48,49] were included for sensitivity when investigating only pancreatic masses, of which six [33,44,45,46,48,49] were used for the combined methods, seen in Supplementary Figure S1. For conventional smear, sensitivity was 0.714 (CI: 0.629–0.787, I^2^: 82%); for LBC, it was 0.747 (CI: 0.643–0.828, I^2^: 84.1%); and for the combination, sensitivity was 0.862 (CI: 0.824–0.893, I^2^: 52.7%). The difference between conventional smear and LBC was not significant (*p* = 0.5942). The difference between conventional smear/LBC and the combination was significant (*p* = 0.001).

**Figure 2:**
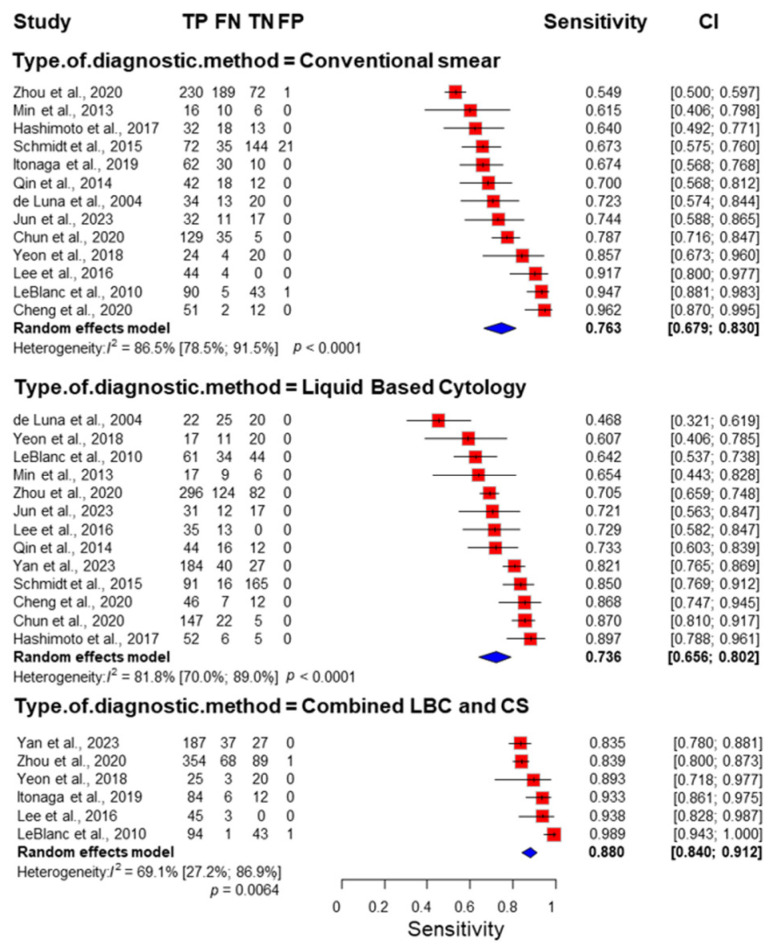
Forest plot representing the sensitivity of different cytology methods [25,27,32,33,36,38,41,42,45,46,47,48,49]. The difference between conventional smear and LBC was not significant (*p* = 0.611). The difference between conventional smear/LBC and the combination was significant (*p* = 0.001/*p* = 0.006). TP: True positives, FN: false negatives, TN: True negatives, FP: False positives. CI: Confidence interval, LBC: liquid-based cytology, CS: conventional smear.

Thirteen studies [25,27,32,33,36,38,41,42,45,46,47,48,49] were included for sensitivity when investigating all abdominal masses, of which six [33,44,45,46,48,49] were used for combined methods, seen in Figure 2. For conventional smear, sensitivity was 0.763 (CI: 0.679–0.830, I^2^: 86.5%); for LBC, it was 0.736 (CI: 0.656–0.802, I^2^: 81.8%); and for the combination, sensitivity was 0.880 (CI: 0.840–0.912, I^2^: 69.1%). The difference between conventional smear and LBC was not significant (*p* = 0.611). The difference between conventional smear/LBC and the combination was significant (*p* = 0.001/*p* = 0.006).

**Figure 3:**
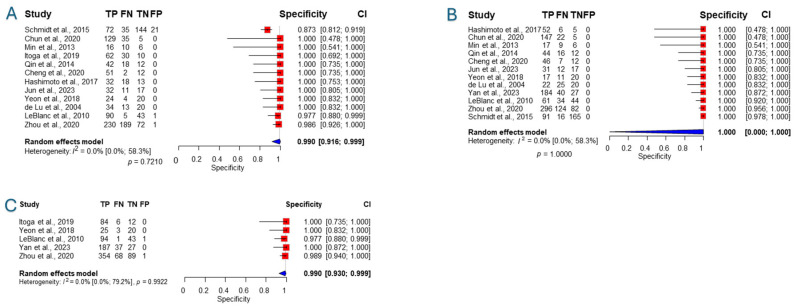
Forest plot representing the specificity of different cytology methods for all abdominal masses [25,27,32,33,36,38,41,42,45,46,47,48,49]. (**A**): Conventional Smear, (**B**): Liquid-based cytology, (**C**): Combination. TP: True positives, FN: false negatives, TN: True negatives, FP: False positives. CI: Confidence interval.

The same thirteen studies also reported data for calculating specificity for all abdominal masses. Nearly all studies showed 100% specificity with no cases of false positives. Notable exceptions were Schmidt [42] with 21 false positives, LeBlanc [48] with 1, and Zhou with 1 [46]. We present in Figure 3 visualizations of the specificities.

Three of the included studies were conference abstracts. To mitigate potential bias, the analysis was run omitting these three studies (Supplementary Figure S2). For conventional smear, sensitivity was 0.761 (CI: 0.665–0.836, I^2^: 88.1%); for LBC, it was 0.719 (CI: 0.618–0.803, I^2^: 83.3%); and for the combination, sensitivity was 0.881 (CI: 0.839–0.913, I^2^: 69.1%).

### 3.4. Accuracy

The pooled sensitivity and specificity results were used to calculate accuracy. The accuracy of CS was 93.12% (actual w = 0.723), that of LBC was 93.45% (actual w = 0.739), and that of the combination was 97.44% (actual w = 0.805). The accuracy for w = 0.2, w = 0.4, w = 0.6, w = 0.8 and w = 1.0 is shown in Supplementary Table S1.

One study [43] was included in this review, which was not included in sensitivity and specificity analysis but reported accuracy. It reported that the accuracy for malignancy was 66.20% (47/71) for conventional smear and 81.70% (58/71) for liquid-based cytology.

### 3.5. Inadequacy Rate

**Figure 4:**
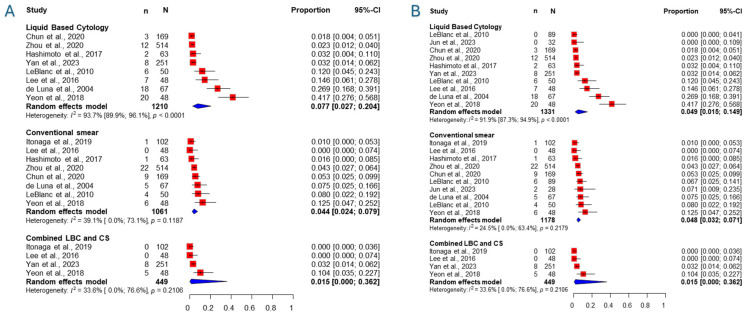
Forest plot representing the rate of inadequate samples of different cytology methods [27,32,33,36,44,45,46,47,48,49]. (**A**): Pancreatic masses, (**B**): All abdominal masses. N: Number of samples, n: number of inadequate samples, CI: confidence interval, LBC: liquid-based cytology, CS: conventional smear.

Nine studies [27,32,33,44,45,46,47,48,49] were included for the rate of inadequate samples for pancreatic masses alone, of which four were included for the combination of methods, seen in Figure 4. The inadequacy rate of LBC was 7.7% (CI: 2.7–20.4, I^2^: 93.7%), that of conventional smear was 4.4% (CI:2.4–7.9, I^2^: 39.1%), and that of the combination was 1.5% (CI: 0–36.2%, I^2^: 33.6%).

Eleven studies [27,32,33,36,44,45,46,47,48,49] were included for the rate of inadequate samples for all types of abdominal masses, of which four were included for the combination of methods, seen in Figure 4. The inadequacy rate of LBC was 4.9% (CI: 1.5–14.9, I^2^: 91.9%), that of conventional smear was 4.8% (CI: 3.2–7.1, I^2^: 24.5%), and that of the combination was 1.5% (CI: 0–36.2, I^2^: 33.6%). *p*-values of the comparison of CS to the combination, LBC to the combination, and CS to LBC were 0.063, 0.15, and 0.66, respectively.

Importantly, confidence intervals were very wide, and differences between interventions should be considered hypothesis-generating.

### 3.6. Risk of Bias Assessment

**Figure 5:**
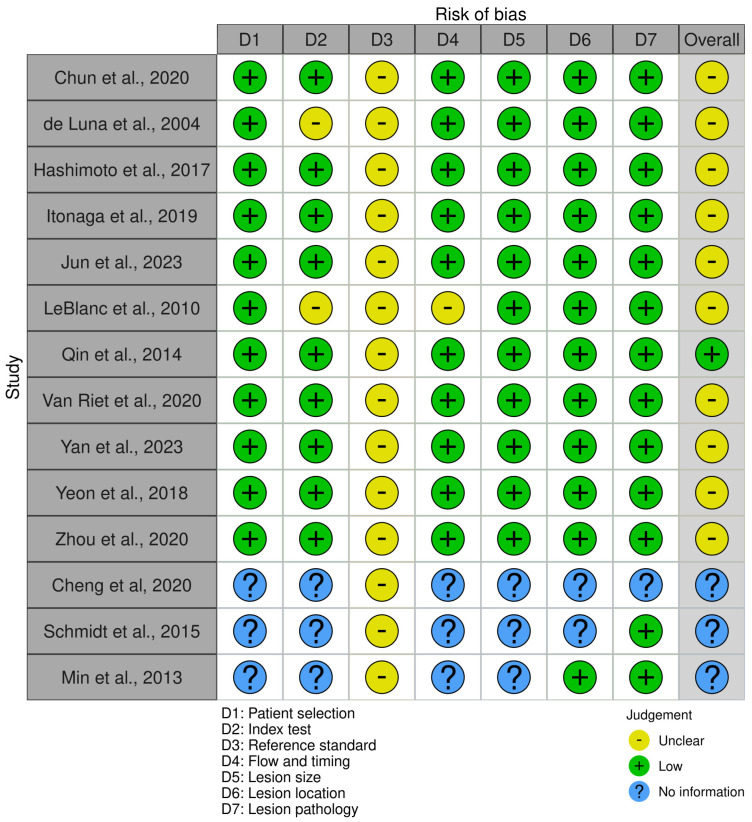
Result of the risk of bias assessment [25,26,32,33,36,38,41,42,43,44,45,46,47,48].

**Figure 6:**
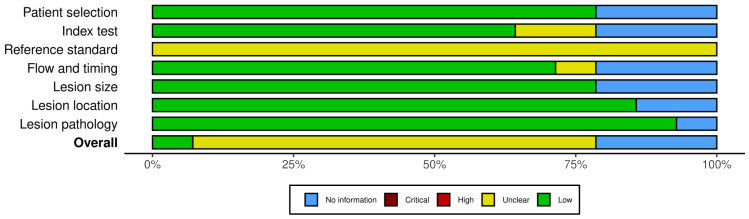
Overall result of the risk of bias assessment.

The results of the risk of bias assessment are found in Figure 5, and a summary can be found in Figure 6. Most studies were found to have a moderate risk of bias overall, primarily due to an impaired domain 3 (reference domain). Due to the inclusion of the cytology result in the reference test, there is a significant risk of incorporation bias in most studies, with a moderate risk of impacting results. Three studies were classified mostly as “No information” due to being conference abstracts. To truly understand the results in context of the risk of bias, the analysis excluding conference abstracts could be referred to.

### 3.7. Publication Bias and Heterogeneity

Peters’ test for publication bias was not statistically significant; details can be found in the Supplementary Material (Supplementary Figures S3 and S4).

## 4. Discussion

In this systematic review and meta-analysis, we evaluated all available studies that directly compared LBC and CS for EUS-guided tissue acquisition from abdominal and pancreatic masses, as well as studies assessing their combined use. While LBC and CS performed similarly overall, their combination significantly improved sensitivity. Specificity was uniformly high across all methods, without notable differences observed. Furthermore, CS demonstrated a slightly lower inadequacy rate than LBC in pancreatic masses, while combining the two methods substantially reduced the rate of inadequate samples.

It is well known that performing multiple passes during EUS-guided tissue acquisition can increase sensitivity. Some of the included studies that assessed the combined use of CS and LBC obtained one pass per method, meaning that the combination group had a total of several passes while the single-method groups had only one—a potential confounder. However, among these studies, those using this approach (Yan and Lee) did not show the highest sensitivity, with one ranking last and the other second among the studies included. Another study (Yeon) performed five passes per method but ranked only fourth in sensitivity. The above observations suggest that the diagnostic benefit of combining CS and LBC is likely independent of the number of passes.

Our analysis confirmed that LBC and CS yield comparable results, but their combination improves diagnosis. As expected, combining methods can only enhance sensitivity, yet the observed difference was both statistically significant and clinically important—yielding nearly a 10% increase in sensitivity without any reduction in specificity. This result demonstrates that a nondiagnostic result from one method does not predict a nondiagnostic result from the other, as they may be able to diagnose different subsets of cases.

Specificity was uniformly high in all studies, with few or no false positives. However, this may be partly due to study design. Many included studies used a composite reference standard that incorporated surgical pathology, biopsy results, clinical follow-up, and, in some cases, cytology itself. While combining multiple confirmation methods and including follow-up strengthens diagnostic accuracy, including the index test in the reference standard introduces incorporation bias. This issue was reflected in our risk of bias assessment, where most studies were rated as having moderate risk due to reference standard limitations. However, the main impact of this incorporation bias would be on false positives and specificity, which we chose to only review in this paper.

We also analyzed the inadequacy rates for each method. When pooling data from all abdominal masses, CS and LBC performed similarly, with inadequacy rates of 4.8% and 4.9%, respectively. In pancreatic masses, however, LBC showed a slightly higher inadequacy rate (7.7%) than CS (4.4%). Although confidence intervals were wide and statistical significance was not reached, four studies (de Luna, Lee, LeBlanc, and Yeon) reported higher inadequacy rates with LBC. These studies used various LBC platforms (PreservCyt, ThinPrep, CellPrepPlus), which were also used in other studies with better results. Notably, three of the four were among the earliest studies included (published in 2004, 2010, and 2016), and while Yeon was published more recently, patient enrollment ended in 2013. LBC as a method for non-gynecological cases was first introduced by a single publication in 1996 [51], followed by one further in 2002 [52]. The method has since been under development. It is possible that early user inexperience may have contributed to higher inadequacy rates for LBC in these studies, which resolved with time.

We also assessed the inadequacy rate of the combined CS and LBC method, which was markedly lower at 1.5%. Although this difference was not statistically significant, it is clearly of clinical relevance—representing a threefold reduction compared to either method alone. As with sensitivity, the potential confounding effect of multiple passes was considered. Nevertheless, studies using multiple passes for the combined method (Yan, Lee) did not show the best performance, while the study with five passes per method had the highest inadequacy rate (10.4%). This finding further supports the interpretation that the diagnostic advantage of combining CS and LBC is genuine and not solely due to the sampling technique. A sample deemed inadequate by one method may still be diagnostic when processed by the other.

We identified three previous meta-analyses on the topic. Zhang et al. [9] assessed the use of LBC and CS (with ROSE) in pancreatic masses and concluded that CS was the superior method in terms of sensitivity (78%, CI: 67–87%) over LBC (75%, CI: 67–81%), while finding, similarly to our investigation, a specificity of 100%. Chandan et al. [7] compared CS to precipitation-based (SurePath) and filtration-based (ThinPrep) LBC for pancreatic masses and concluded that precipitation-based LBC was the superior method in terms of sensitivity (79.2%, CI: 70.7–85.7%; 83.6%, CI: 70.7–91.5%; and 68.3%, CI: 55.3–79%, respectively). Pan et al. was able to compare these two methods to a combined approach based on 8 studies, finding a higher sensitivity for LBC than for CS (76%, CI: 72–79% vs. 68%, CI: 64–71%), and a superior sensitivity still for the combination (87%, CI: 84–90%). Overall, our results are similar for the combination at 89%; however, the 74% and 75% sensitivities for CS and LBC demonstrate the relative comparability of the two methods.

There was some clinical heterogeneity among the included studies. For one, the included studies used different needle sizes, with some limiting themselves to 22G or 20G needles, and others using a mix of other available needle sizes. Needle size has been investigated previously as impacting sensitivity of tissue acquisition, although a meta-analysis investigating different needle sizes for FNA [53] did not find a difference in sensitivity or adequacy between 22G and 25G needles. Regardless, there is a potential that needle size may impact the specific performance of one cytology method over the other.

Prior reviews indicate that ROSE materially modifies cytology performance and helps explain why meta-analyses have disagreed on the “preferred” method. Zhang et al. concluded that smear cytology with ROSE yields the highest diagnostic performance, recommending LBC primarily when ROSE is unavailable, whereas Chandan et al. emphasized that in no-ROSE settings, certain LBC platforms (e.g., precipitation-based) can outperform smears; Pan et al. did not stratify by ROSE but showed a clear incremental gain when combining LBC and smears [7,8,9]. In our dataset, ROSE use was inconsistently reported, precluding a formal moderator analysis; nevertheless, the consistently superior sensitivity of the combined CS+LBC strategy and its lowest inadequacy rate suggest that using both preparations likely mitigates ROSE-related variability—enhancing yield when ROSE is present (by capturing discordant positives) and partially compensating when ROSE is absent (by reducing nondiagnostic samples).

Current ESGE guidelines [10] recommend preparing EUS-guided tissue acquisition samples using either LBC alone or a combination of CS and LBC, depending on local expertise. This recommendation is weak and based on limited evidence. Our findings support the combined use of CS and LBC, further suggesting that if only one method is selected, CS might be the preferred option to minimize the risk of inadequate samples.

### 4.1. Strengths and Limitations

In the performance of this study, we aimed to include only studies that allowed us to compare cytology methods directly, and we were able to include a great number of patients.

This study is limited somewhat by clinical heterogeneity among the studies, particularly in needle types, staining, and processing—all of which are known to impact performance. Additionally, articles were published across two decades (2004–2023), during which time cytology technology improvements may have introduced temporal bias. This was also reflected in substantial heterogeneity. For inadequacy rates, CIs were wide, particularly for inadequate samples, limiting interpretation.

### 4.2. Implication for Practice and Research

To translate these findings into clinical benefit [54,55], we recommend that both CS and LBC be used following EUS-guided tissue acquisition to improve diagnostic sensitivity and reduce the rate of inadequate samples. Current guidelines should be updated accordingly. Further studies are needed to confirm the observed reduction in inadequacy rates.

## 5. Conclusions

A combination of CS and LBC significantly increases sensitivity after EUS-guided tissue acquisition for abdominal masses, with a clinically important difference.

## Figures and Tables

**Table 1:** Basic characteristics of included studies. N: Number of patients, CS: Conventional Smear, LBC: Liquid-based cytology, EUS: Endoscopic ultrasound, FNA: fine needle aspiration, IQR: Interquartile range, NA: Not available, HE: Hematoxylin/Eosin, PSM: Propensity score matched. * Conference abstracts.

Study	Study Design	Study Period	Country	Population	Mass	Needle Type	Reference	Description of Assessments	Staining	ROSE	Sample
Chun 2020 [27]	Randomized, crossover	April 2018–March 2019	Republic of Korea	N: 170, Female: 44.1%, age: 64.8 ± 10.6 (37–88)	Pancreatic	19/22G EZ Shot 3 Plus (Olympus Medical Systems, Tokyo, Japan)	LBC, CS; EUS-FNA core biopsy or surgical specimen, 6-month clinical/radiological follow-up	CS: alcohol, LBC: CytoRich Red, SurePath	Papanicolaou	Unclear/No	Individual
de Luna 2004 [47]	Retrospective, crossover	August 2000 to February 2002	USA	N: 67, Female: 32.8%, age: 64 (39–87)	Pancreatic	NA	Histologic and clinical follow-up	CS: alcohol/air, LBC: PreservCyt, ThinPrep	Modified Giemsa for air-dried smears, Papanicolaou for alcohol-fixed and LBC samples	Unclear	Split
Hashimoto 2017 [32]	PSM	January 2009–August 2014	Japan	N: 126, Female: 49.2%, age: CS: 65 (35–93), LBC: 66 (33–85)	Pancreatic	19G/22G/25G	Surgical resection, additional EUS-FNA procedure, 6 months clinical/imaging follow-up	CS: alcohol, LBC: SurePath	HE for smears, Papanicolaou for LBC samples	No	Individual
Itonaga 2019 [33]	PSM	December 2011 to October 2017	Japan	N: 311, Female: 42.8%, age: NA	Pancreatic	19G/22G/25G Expect (Boston Scientific) or EZ Shot2 (Olympus Medical)	Surgical resection, 12-month clinical follow-up	LBC: ThinPrep, CS: Air/alcohol	Air-dried: Diff-quick, alcohol-fixed and LBC Papanicolaou	Yes	Split
Jun 2023 [36]	Randomized crossover	January 2019 to August 2022	Republic of Korea	N: 60, Female: 53.3%, age: 60.7 ± 12.8 (24–85)	Abdominal	19G/22G EZ Shot 3 Plus (Olympus)	Core biopsy, LBC; CS and surgical specimens, plus 6-month follow-up	LBC: CytoRich, SurePath vials, PrepStain processor, CS: alcohol	NA	No	Individual
LeBlanc 2010 [48]	Prospective crossover	April 2005 through April 2007	USA	N: 50, Female: 36% (whole population), age: mean 63	Subepithelial masses	22G EUSN-3 or echo-1-22	Final cytological diagnosis, surgical pathology, or follow-up	CS: air/alcohol, LBC: ThinPrep	Air-dried: Diff-quick, alcohol-fixed and LBC Papanicolaou	Yes	Individual
van Riet 2010 [43]	Prospective crossover	April 2016–2017	The Netherlands	N: 71, Female: NA, age: NA	Solid pancreatic lesions	19G, 22G, 25G Echotip/Expect	Surgical resection, 12-month clinical follow-up	CS: no information, LBC: Thinprep	Different per center: Hemocolor, no stain, Diff quick, or Giemsa	No	Split
Yan 2023 [44]	Retrospective crossover	January 2014 to February 2022	China	N: 251, Female: 41%, age: Median 60 (IQR: 13)	Pancreatic	20G FNA	Needle biopsy/surgery sample, FNA sample, 6-month clinical/imaging follow-up	CS: alcohol, LBC: ThinPrep	NA	Not all	Individual
Yeon 2018 [45]	Prospective crossover	June 2012–October 2013	Republic of Korea	N: 43, Female: 32.6%, age: 65.5 ± 12.5	Pancreatic	22G Echotip	Biopsy, surgery, or 6-month clinical follow-up	CS: 95% alcohol fixation, LBC: CellPrepPlus	NA	Unclear	Individual
Zhou 2020 [46]	Retrospective crossover	January 2015 to January 2019	China	N: 514, Female: 37.2%, age: Median 60 (IQR 50–67)	Pancreatic	22G/25G	FNA, surgical pathology, 6-month clinical follow-up	CS: alcohol, LBC: CytoRich	CS: HE, LBC: Papanicolaou	Not all	Split
Qin 2014 [41]	Prospective crossover	January 2011 to January 2014	China	N: 72, Female: 19.4%, age: 54.6 (24–70)	Pancreatic	22G FNA	Surgery, 9-month clinical follow-up	CS: alcohol/air, LBC: ThinPrep	Modified Diff-Quick, Papanicolaou	No	Individual
Cheng 2020 [25] *	NA	December 2016 to January 2018	China	N: 52, Female: NA, age: NA	Abdominal	NA	Pathology, follow-up	NA	NA	NA	NA
Schmidt 2015 [42] *	NA	2008 to 2011	Germany	N: 172, Female: 39%, age: 64.8 (±12.4)	Pancreatic	NA	Surgical histology, 12-month follow-up	NA	NA	NA	NA
Min 2013 [38] *	prospective crossover	November 2010 to February 2013	China	N: 32, Female: NA, age: NA	Pancreatic	NA	NA	NA	NA	NA	NA
Lee 2016 [49]	Retrospective crossover	July 2010 to June 2015	Republic of Korea	N: 48, Female: 50%, age: Median 67, range 39–84	Pancreatic	22G FNA	Clinical and imaging follow-up of 12 months, CS, LBC, and pathology from metastatic sites	CS: alcohol, LBC: ThinPrep	Papanicolaou	No	Individual

## Data Availability

The datasets used in this study can be found in the full-text articles included in the systematic review and meta-analysis. If further information is needed, it will be provided upon reasonable request to the corresponding author.

## Supplementary Materials

The following supporting information can be downloaded at: https://www.mdpi.com/article/10.3390/jcm14186685/s1, Supplemental Text, Figure S1: Forest plot representing the sensitivity of different cytology methods in pancreatic masses; Figure S2: Forest plot representing the sensitivity of different cytology methods in all abdominal masses, excluding conference abstracts; Figure S3: Funnel plot for publication bias of liquid based cytology; Figure S4: Funnel plot for publication bias of smear cytology; Table S1: Estimated accuracy for different values of w (disease prevalence).
