# Supplementary material for: Combined Liquid-Based Cytology and Conventional Smear Provides Better Sensitivity and Adequacy Rates After Endoscopic Ultrasound-Guided Tissue Acquisition of Abdominal Masses: A Systematic Review and Meta-Analysis
Source: J Clin Med. 2025 Sep 22;14(18):6685. doi: 10.3390/jcm14186685 (PMC12471235; doi:10.3390/jcm14186685)
Supplement: Supplementary file 1 [file jcm-14-06685-s001.zip › jcm-3826037-supplementary.pdf]

# Supplementary Material

**Text S1 Search strategy:**

PubMed:

(pancrea* OR gastrointest* OR abdom*) AND (FNA OR FNB OR (tissue AND acquisition) OR (fine AND needle)) AND ("tissue preparation" OR (smear AND “liquid cytology"[tiab:~4]))

Embase:

(pancrea* OR gastrointest* OR abdom*) AND (FNA OR FNB OR (tissue AND acquisition) OR (fine AND needle)) AND ("tissue preparation" OR (smear AND (liquid NEAR/4 cytology))

CENTRAL:

(pancrea* OR gastrointest* OR abdom*) AND (FNA OR FNB OR (tissue AND acquisition) OR (fine AND needle)) AND ("tissue preparation" OR (smear AND (liquid NEAR/4 cytology))

## Figures

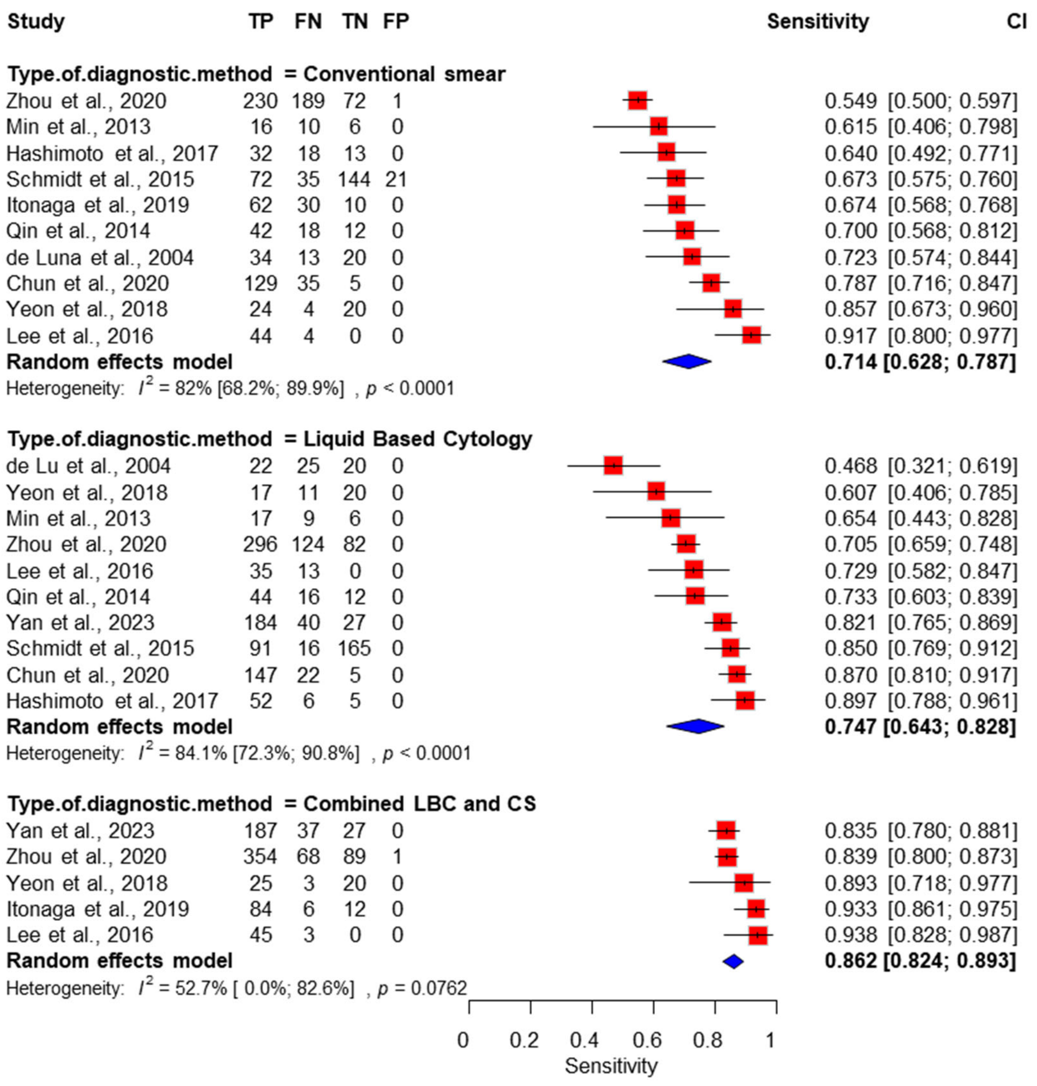


**Supplementary figure S1:** Forest plot representing the sensitivity of different cytology methods in pancreatic masses[25,27,32,33,38,41,42,45–49]. TP: True positives, FN: false negatives, TN: True negatives, FP: False positives. CI: Confidence interval, LBC: liquid-based cytology, CS: conventional smear.

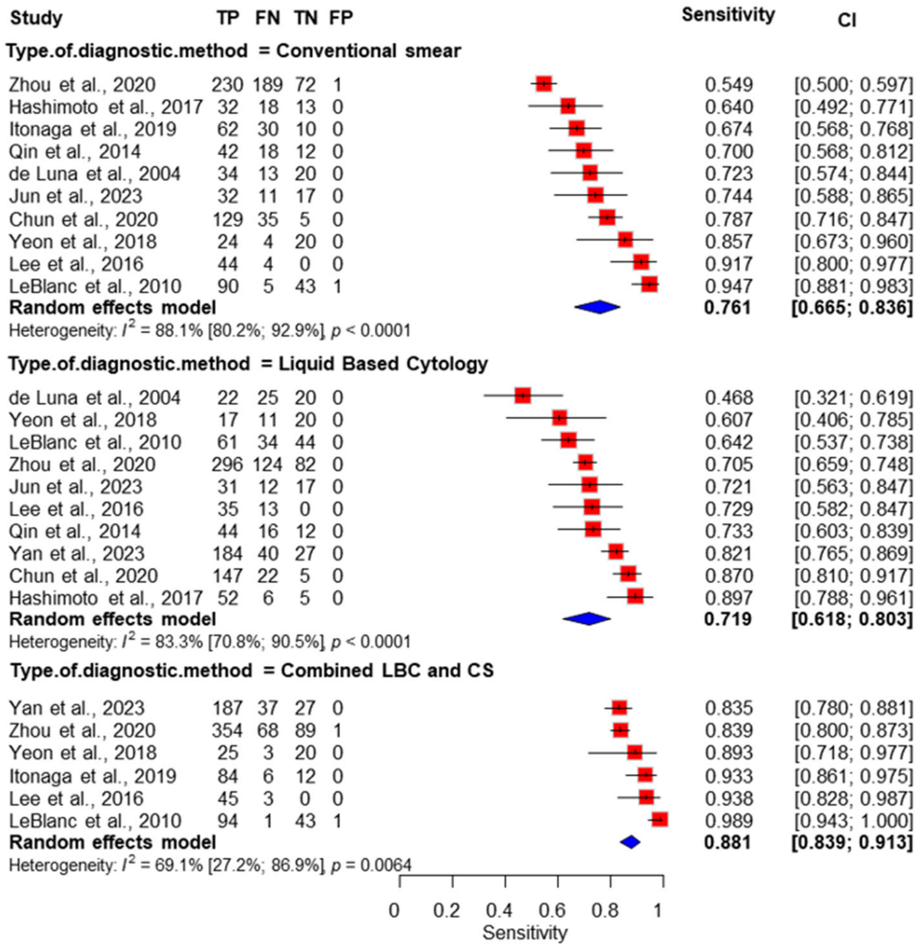


**Supplementary figure S2:** Forest plot representing the sensitivity of different cytology methods in all abdominal masses[27,32,33,36,41,45–49], excluding conference abstracts.

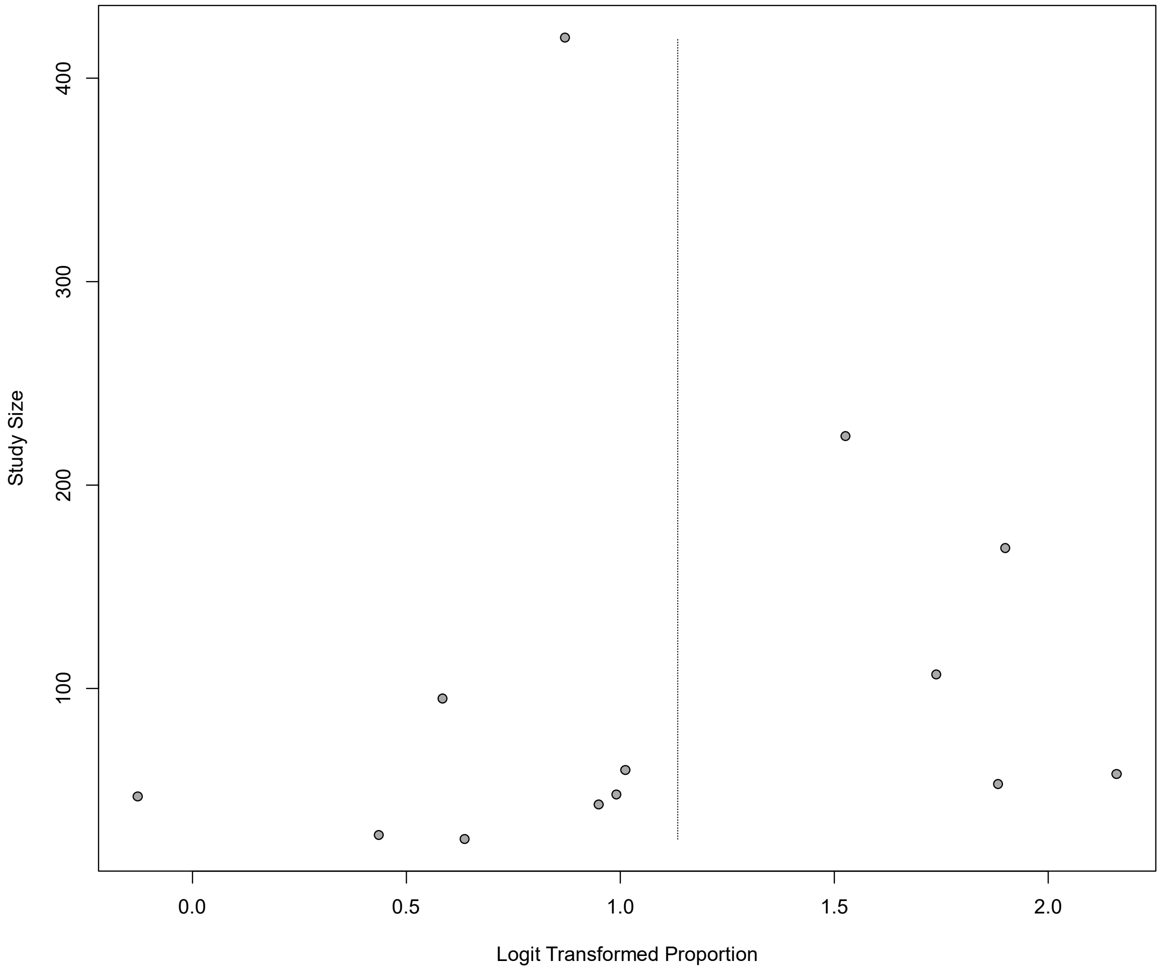


**Supplementary Figure S3:** Funnel plot for publication bias of liquid based cytology. Linear regression test of funnel plot asymmetry. Test result: t = -0.70, df = 11, p-value = 0.4988. Bias estimate: -11.5416 (SE = 16.5013). Details: multiplicative residual heterogeneity variance (tau^2 = 5.9087), predictor: inverse of total sample size, weight: inverse variance of average event probability

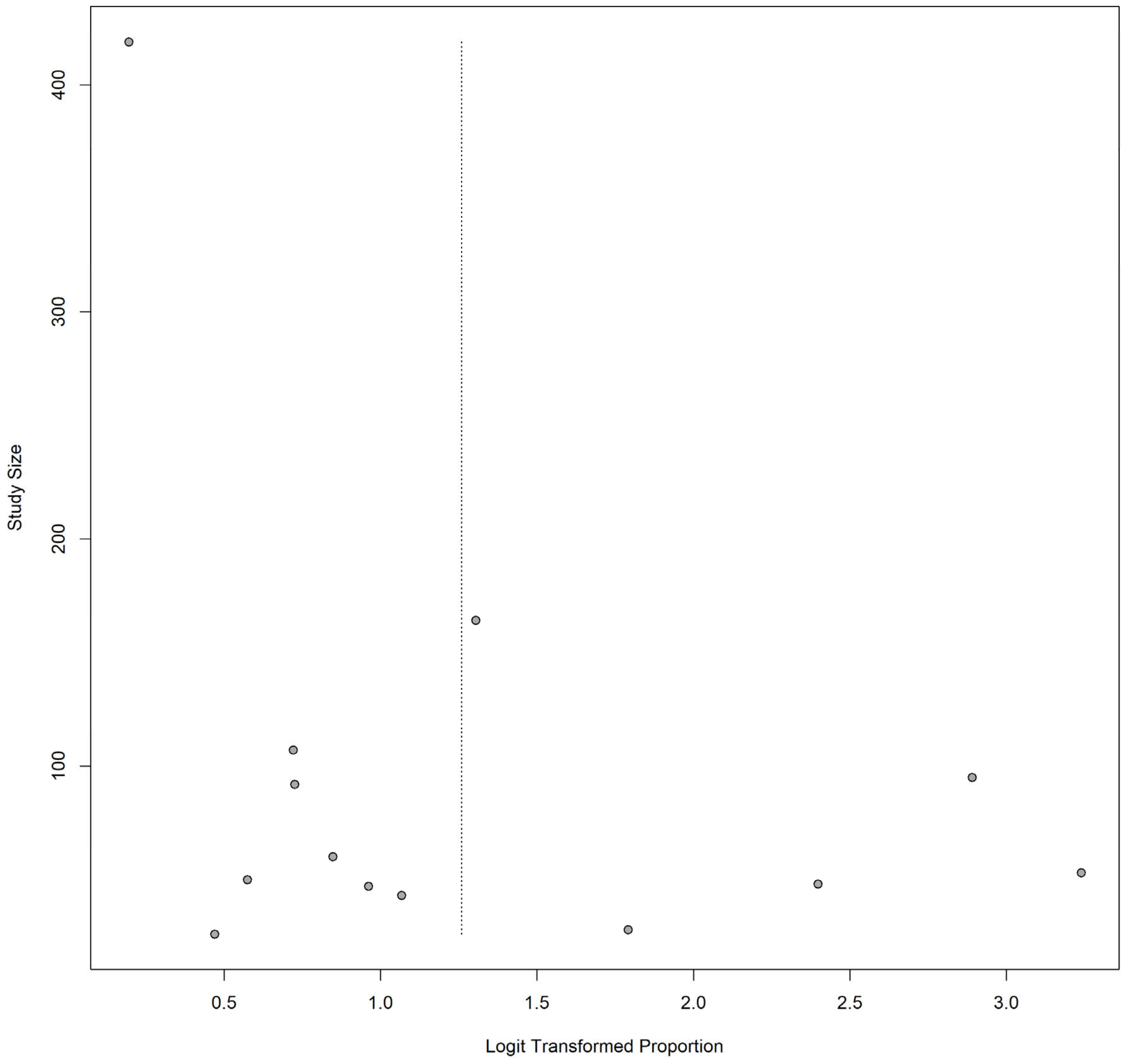


**Supplementary Figure S4:** Funnel plot for publication bias of smear cytology. Linear regression test of funnel plot asymmetry. Test result: t = 1.77, df = 11, p-value = 0.1037, Bias estimate: 32.5441 (SE = 18.3454). Multiplicative residual heterogeneity variance (tau^2 = 6.5234). Predictor: inverse of total sample size. Weight: inverse variance of average event probability

**Supplementary Table S1:** Estimated accuracy for different values of w (disease prevalence):

| **Method** | **$N_{Benign}$** | **$N_{malignant}$** | **w** | **Accuracy** | **Accuracy (w=0.2)** | **Accuracy (w=0.4)** | **Accuracy (w=0.6)** | **Accuracy (w=0.8)** | **Accuracy (w=1.0)** |
|---|---|---|---|---|---|---|---|---|---|
| Combination | **188** | **775** | 0.80 | 0.97 | 0.90 | 0.93 | 0.95 | 0.97 | 1.00 |
| CS | **444** | **1156** | 0.72 | 0.93 | 0.82 | 0.86 | 0.90 | 0.95 | 0.99 |
| LBC | **458** | **1295** | 0.74 | 0.93 | 0.79 | 0.85 | 0.90 | 0.95 | 1.00 |
